# Vehicle logistics intermodal route optimization based on Tabu search algorithm

**DOI:** 10.1038/s41598-024-60361-7

**Published:** 2024-05-24

**Authors:** Siyuan Ru

**Affiliations:** Graduate School of Business, University Kebangsaan Malaysia, 43600 Bangi, Selangor Malaysia

**Keywords:** Tabu search algorithm, Vehicle logistics, Intermodal route optimization, P-median model, Graph traversal algorithm, Engineering, Mathematics and computing

## Abstract

With the development of logistics enterprises and the adjustment of some relevant laws and regulations, the profit space of vehicle logistics enterprises has been further compressed. To reduce vehicle logistics transportation cost and increase the profit space of vehicle logistics, the vehicle logistics multimodal transport network is constructed and the graph traversal algorithm is used to screen the feasible paths in the vehicle logistics multimodal transport network. Then, the Tabu search algorithm can optimize vehicle logistics multimodal transport route model. Results showed that Tabu search performed better than other methods in solving route optimization problems. The cost of Tabu search algorithm after convergence was 1.2 yuan/km × per set. The performance of Tabu search algorithm on NGSIM data set was better than other methods. On this data set, the area under the curve of Tabu search algorithm was much higher than that of other methods. The optimization results of Tabu search for vehicle logistics intermodal routes were effective. Among the 15 routes, only four routes were not optimized, and other routes were optimized. After optimization, the profits have increased, and the profit of Route 9 had the largest increase, which was 18%. The research successfully constructs the optimization model of vehicle logistics intermodal route, and completes the solution to increase the profit space of vehicle logistics enterprises.

## Introduction

With the development of the Internet, people's shopping methods have gradually diversified and are no longer limited to offline physical stores. More and more people are choosing online shopping, and online shopping cannot do without the support of efficient logistics systems. In addition to personal online shopping, many cross provincial and city company trade also relies on the support of logistics systems^1,2^. Automotive logistics, as an important component of the logistics system, plays a key role in vehicle logistics. Automotive logistics is built on top of road transportation, but due to some regulatory adjustments, the single vehicle transportation volume of road transportation has decreased, further compressing the profit space of logistics companies. In order to improve the profit margin of logistics companies and reduce logistics transportation costs, we need to increase logistics transportation methods^3,4^. Common logistics transportation methods include not only road transportation, but also railway transportation and waterway transportation. By changing the transportation mode of vehicle logistics and adding railway and waterway transportation to traditional road transportation, the advantages of various transportation methods can be fully utilized, which can effectively improve the profit space of logistics enterprises. In the entire vehicle logistics system, adding railway and waterway transportation will reduce the transportation time and cost of the logistics system. However, an unreasonable combination of transportation methods may actually increase transportation time and costs. Therefore, in order to improve the profit margin of vehicle logistics companies, this study adopts taboo search algorithm to reasonably match the transportation and distribution methods of the logistics system, optimize the proportion of various transportation methods in vehicle logistics, and thereby reduce the transportation cost of vehicle logistics. The research innovatively proposed an optimization method for vehicle logistics transportation mode based on taboo search algorithm. By using taboo search algorithms, the proportion of various transportation methods in vehicle logistics can be reasonably arranged, taking into account factors such as transportation time and cost. This method can effectively reduce the transportation cost of vehicle logistics and improve the profit margin of logistics enterprises. The first part is a summary of the research status of vehicle logistics and vehicle logistics. The second part is the application of Tabu Algorithm in vehicle logistics. The third part analyzes the simulation results of model built in the research. The fourth part is a summary of the research content.

## Related works

Logistics system is the basis of cross regional transactions. In order to solve the problem of path planning for green vehicles in cold chain logistics, Li et al. established a vehicle logistics path optimization model. Results showed that this algorithm can reduce the total cost of the full set of room temperature gas emission logistics system and reduce greenhouse gas emissions within a certain range. However, the focus of the model is to reduce the emissions of the full set of room temperature gas emission cold chain logistics system, rather than increase the profit space of logistics enterprises^5^. Maiyar et al. built a sustainable grain transportation model to solve the problem of grain transportation in the middle of the hub between Indian states. The model comprehensively considered the transportation cost, hub re-selection, route change, and other issues, and focused on reducing the grain transportation cost. The results showed that when the single hub was interrupted, the cost increased by 14%, which can meet the food demand, and when the multi-hub was interrupted, the cost increased by 40%, which can meet the demand. This model only served the purpose of reopening grain transportation and did not reduce transportation costs^6^. To improve passengers’ transportation efficiency under multiple modes cooperation of transportation, Butko et al. proposed a genetic algorithm integrating factor analysis method to improve the passenger transportation technology and route. Among them, factor analysis method was used to analyze the main reasons for passengers' choice of transportation mode, and genetic algorithm was used to optimize the coordination between various transportation modes. The results showed an effective efficiency improvement of train transportation and passenger transportation. However, this method considered less intermodal modes and focused on train transportation^7^. To improve transportation efficiency of highway railway intermodal transport, Y Sun established a highway railway intermodal route optimization model, which comprehensively considered the factors such as transportation capacity, travel time, loading and unloading time and train departure time, and expressed uncertain parameters and variables. Results showed that the model can obtain a reliable green path, but the final optimization result was to reduce the pollution mitigation in the transportation process, not improve the transportation efficiency, or reduce the transportation cost^8^. In order to develop the fourth logistics, W Hong et al. established the multi-objective optimization model of the fourth logistics, and proposed the enhanced Elite Genetic Algorithm for the fourth logistics path optimization. Results showed that this algorithm can solve the path optimization problem of the fourth party logistics, but the optimization effect of this method was poor^9^.

Tabu search algorithm is a commonly used path optimization algorithm. To optimize the power curve of wind turbine, Karamichailidou et al. proposed radial basis function neural network to optimize the Tabu search, and used this optimized algorithm to improve modeling accuracy of wind turbine power curve. Results showed that the method proposed by the author can effectively improve curve accuracy^10^. To optimize the storage address of the energy storage unit in the Internet system, Chen et al. proposed a location analysis method using Tabu search algorithm, which took the network loss as the constraint condition of location. The results showed that Tabu search algorithm can effectively find the optimal node solution in the location of energy storage units on the Internet, which was feasible for the location analysis of energy storage units^11^. In order to increase the security of secret files, Rao et al. proposed an image encryption method using pixel expansion threshold, and used Tabu search to improve this method. Results showed that this algorithm can effectively solve complex combination problems^12^. Maha· et al. proposed a Tabu search with time window to optimize the path at different time points in urban tourism in order to improve the vehicle path planning problem in urban tourism road network. Results showed that this algorithm can improve the time spent on the road by customers in urban tourism and improve the efficiency of customers' urban tourism^13^.

To sum up, path optimization has always been a key research direction in the logistics field and individual fields, but most path optimization only considers the impact of environmental pollution, and does not take reducing the transportation cost as the main consideration direction. Tabu search algorithm is a commonly used path optimization algorithm. Therefore, the study proposes to take the logistics transportation cost as the constraint condition of Tabu search algorithm to optimize the vehicle logistics intermodal route, so as to reduce logistics transportation costs and increase the profit space of logistics enterprises.

## Application research of Tabu search algorithm in vehicle logistics intermodal route optimization

Tabu search algorithm can effectively solve various path optimization problems. Therefore, the research content of this chapter is Tabu search, which optimizes vehicle logistics intermodal route. The first section is the optimization of vehicle logistics intermodal route, and the second section is the Tabu search algorithm.

### Vehicle logistics intermodal network structure research

**Figure 1:**
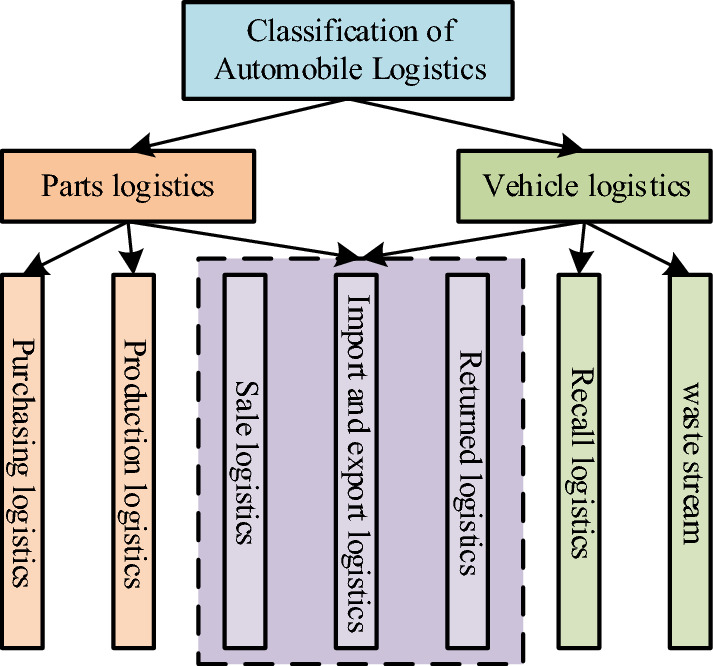
Automotive logistics structure.

Vehicle logistics is a part of vehicle logistics, which refers to the process of delivering vehicles from the plant to the vehicle distribution, and then from the vehicle distribution to the dealers. Automobile logistics includes parts logistics and vehicle logistics. Parts logistics includes procurement, production, sales, recycling, and import and export logistics. Vehicle logistics has one more level than parts logistics. The first level includes waste logistics, recycling logistics, call back logistics, sales logistics and import and export logistics. The second level is subordinate to sales logistics, which is divided into engineering vehicle logistics, commercial vehicle logistics Passenger vehicle logistics and special vehicle logistics are shown in Fig. 1^14,15^.

Vehicle logistics has three characteristics. The first is that the demand for logistics frequency is high, and the batch of single logistics is small. The second is that the distribution route of vehicle logistics is complex, and the third is that the safety requirements are high. The order delivery of complete vehicles can be divided into production driven by forecast and production based on order. The first is the order delivery based on market development trend and customer purchase demand forecast. The second is the delivery mode of production based on customer orders. In order to cope with market changes and reduce inventory costs, manufacturers' orders usually show the first feature. The whole vehicle distribution takes the manufacturer as the starting point, the distribution center as the transit, and the dealers all over the country as the destination. Therefore, the whole vehicle logistics will present the second feature. The third feature is that the car is a single piece of high-value goods. If an accident occurs in the distribution process, resulting in the damage of the whole vehicle, it will cause huge economic losses. The transportation mode of vehicle logistics includes railway transportation, waterway transportation and road transportation. The distribution mode is divided into hierarchical distribution mode and manufacturer direct delivery mode. As the vehicle logistics distribution is carried out according to the manufacturer distribution center dealer, the site selection in the distribution is more important. Because the location of the manufacturer and dealer is determined, the research assumes that the distribution center coordinate is $\left(x,y\right)$, the dealer is $\left(j=\text{1,2},...,n\right)$, the plane coordinate is $\left({X}_{j},{Y}_{j}\right)$, and the demand is ${\omega }_{j}$, then the distribution center position can be determined by the gravity model, which is shown in formula (1)^16,17^.

1 $$\left\{\begin{array}{c}x\sum_{j=1}^{n}{\omega }_{j}=\sum_{j=1}^{n}{\omega }_{j}{X}_{j}\\ y\sum_{j=1}^{n}{\omega }_{j}=\sum_{j=1}^{n}{\omega }_{j}{Y}_{j}\end{array}\right.$$

In order to prevent accidents, each distribution center needs to prepare a preparatory coordinate. Formula (2) is the preparatory coordinate.

2 $$\left\{\begin{array}{c}x=\sum_{j=1}^{n}{\omega }_{j}{X}_{j}/\sum_{j=1}^{n}{\omega }_{j}\\ y=\sum_{j=1}^{n}{\omega }_{j}{Y}_{j}/\sum_{j=1}^{n}{\omega }_{j}\end{array}\right.$$

**Figure 2:**
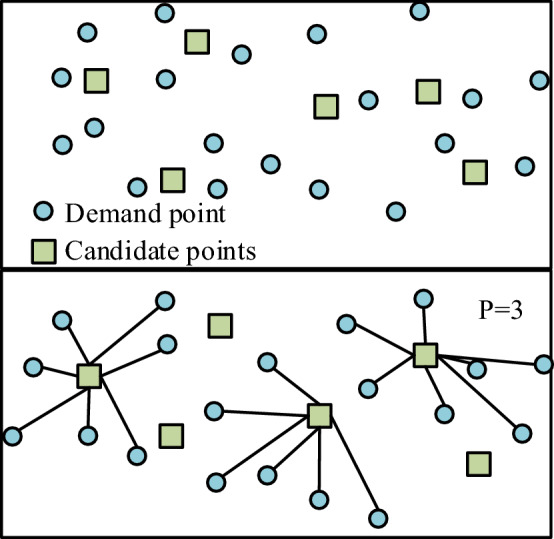
Schematic diagram of p-median model.

In addition to the above mathematical model, the location of distribution centers also needs the help of the traditional *P* median model to complete the establishment of distribution centers with the minimum cost. The encoding method of this model is marked as ‘1’ if the city is selected; Otherwise, it is marked as ‘0’. The model assumes that it is necessary to build *P *new distribution centers, take the dealer as the demand point, set the preparatory coordinates in the alternative centralized city, set the number, location and preparatory coordinates of demand points, make each demand point correspond to a distribution center, and take the minimum cost from the demand point to the distribution center as the target. Then a suitable location is selected for the new distribution center, as shown in Fig. 2.

Using the *P* median model, when selecting the distribution center, only the transportation cost between the distribution center and dealers is considered, and center construction cost and the manufacturer's production capacity are not considered. Therefore, the traditional *P* median model is improved and a new two-level location model is established, as shown in formula (3).

3 $$\text{min}C=\sum_{i}^{I}\sum_{j}^{J}\left({d}_{ij}{s}_{ij}Z\left(i\right){U}_{s}\right)+\sum_{i}^{I}\sum_{j}^{J}\left({d}_{ij}{b}_{ij}Z\left(i\right){U}_{b}\right)+\sum_{i}^{I}\sum_{k}^{K}\left({d}_{ik}{ws}_{ik}Z\left(i\right){U}_{s}\right)+\sum_{i}^{I}\sum_{k}^{K}\left({d}_{ik}{wb}_{ik}Z\left(i\right){U}_{b}\right)+\sum_{i}^{I}\left(Z\left(i\right)Co\left(i\right)\right)$$

In formula (3),${ d}_{ij}$ represents the distance from distribution centerc to dealer $j$, ${s}_{ij}$ is the demand of dealer $j$ for small models in $i$, $Z\left(i\right)$ is used to judge whether the dealer is served by the distribution center $i$. ${U}_{s}$ represents the transportation unit price of small models, ${b}_{ij}$ is the demand of the dealer $j$ for large models in $i$, ${U}_{b}$ is the transportation unit price of large models, ${d}_{ik}$ is the distance from $i$ to manufacturer $k$, and ${ws}_{ik}$ is the distribution of small models in $i$ by $k$, ${wb}_{ik}$ indicates large vehicle types allocation by the manufacturer $k$ to $i$, and $Co\left(i\right)$ indicates the construction cost of the distribution center. In order to ensure that the location of distribution centers is accurate, several constraints are set for the calculation results of formula (3), and the constraints for the number of small models and distribution scheme of dealers are shown in formula (4).

4 $$\sum_{i}^{I}{s}_{ij}={d}_{1}\left(j\right)$$

Formula (5) is the constraints of the number of large models of dealers and the allocation scheme.

5 $$\sum_{i}^{I}{b}_{ij}={d}_{2}\left(j\right)$$

Formula (6) is the constraints on the production capacity of the manufacturer's large vehicles

6 $$\sum_{i}^{I}{wb}_{ik}\le {Pb}_{k}$$

In formula (6), ${Pb}_{k}$ indicates the production capacity of the manufacturer for $k$ large vehicles. See formula (7) for constraints on the production capacity of small cars of manufacturers.

7 $$\sum_{i}^{I}{ws}_{ik}\le {Ps}_{k}$$

In formula (7), ${Ps}_{k}$ indicates the production capacity of $k$ small cars of the manufacturer. See formula (8) for the constraint conditions of the distribution of large models of the manufacturer and the distribution center.

8 $$\sum_{j}^{J}\sum_{i}^{I}{b}_{ij}=\sum_{k}^{K}\sum_{i}^{I}w{b}_{ik}$$

Formula (9) is the constraint conditions of the allocation of small models of the manufacturer and the allocation of the distribution center.

9 $$\sum_{j}^{J}\sum_{i}^{I}{s}_{ij}=\sum_{k}^{K}\sum_{i}^{I}w{s}_{ik}$$

Formula (10) is the constraints of dealer demand and distribution center capacity

10 $$\sum_{j}^{J}\sum_{i}^{I}\left({s}_{ij}+{b}_{ij}\right)\le \sum_{i}^{I}\left(e\left(i\right)Z\left(i\right)\right)$$

**Figure 3:**
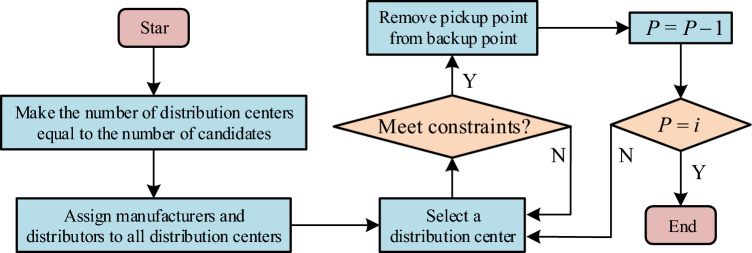
Greek takeover heuristic algorithm calculation steps.

In formula (10), $e\left(i\right)$ indicates the $i$ capacity. After the reconstruction of the distribution neutral light location model, the exact solution cannot be obtained quickly. Therefore, the greedy take away heuristic algorithm can improve distribution center location model. Its goal is to find an equilibrium solution under various comprehensive conditions. Therefore, in this algorithm, the goal and greedy criterion are more important, and can only be used as the optimal choice based on the current basis, the solving steps of the algorithm are relatively simple. The first step is to initialize the parameters so that the number of selected distribution centers is equal to the number of preparatory distribution centers. The second step is to assign manufacturers and dealers to all candidate distribution centers under the condition that formulas (4) to (10) are met. The third step is to determine whether the conditions are met. If yes, a distribution center is taken out. If not, return to the second step, the fourth step is to remove the removed distribution centers from the reserve collection, reduce the number of distribution centers by one, and return to the third step until the number of remaining distribution centers is equal to the number of distribution centers to be selected, as shown in Fig. 3^18^.

### Profit optimal transportation scheme optimization research using Tabu search

**Figure 4:**
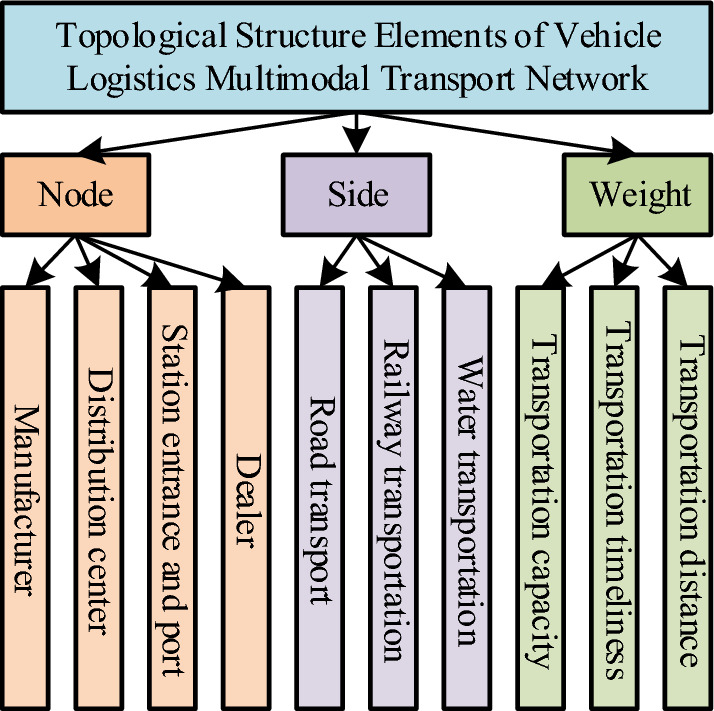
Elements of the topology network for vehicle logistics multimodal transportation.

The optimization of the optimal profit transportation scheme is based on the topology network structure of the vehicle logistics multimodal transport. Therefore, it is necessary to establish the transportation network topology structure first. Manufacturers, distribution centers, transportation nodes and dealers can be used as network nodes to build the vehicle logistics multimodal transport topology network. Its construction needs the location information of dealers, manufacturers and distribution centers; the location relationship of each station and port corresponding to the mode of transportation; Location information of distribution center, port and station entrance; quantity information of local distribution centers, dealers, ports and stations; Storage capacity information of local distribution centers, stations and ports. The network mainly includes three element points: node, edge and weight. Each element is composed of multiple parts. The node element is composed of manufacturers, distribution centers, railway stations, water ports and dealers. The edge element is composed of highway transportation lines, railway transportation lines and water transportation lines. The weight element is composed of transportation volume, transportation time and transportation distance, as shown in Fig. 4.

**Figure 5:**
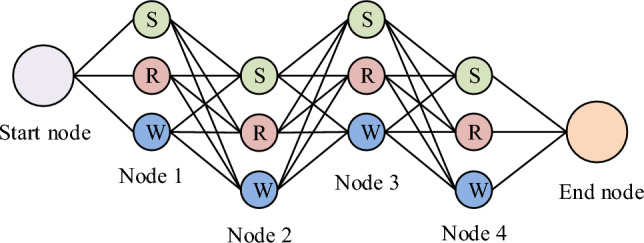
Topological network structure of vehicle logistics multimodal transportation.

The vehicle logistics multimodal transport topology network is constructed according to the idea of graph theory. There are different modes of transport between multimodal transport nodes, and the mode of transport at a node may also change. If only the idea map is used to represent different modes of transport, the attributes of nodes with different modes of transport will be the same, which is different from the actual situation. Therefore, when constructing the multimodal transport network. A single node needs to be expanded and divided into three nodes with different attributes. The expanded nodes have different transportation modes and node attributes, which is more consistent with the reality. The expanded node can be connected with other transportation nodes, but there is no edge between the three nodes after the expansion of the same node. During the expansion process, the starting point of the route, like other nodes, needs to be expanded into three nodes, and the end point is not expanded. The starting point shows that the expanded three nodes are connected with the starting point, and the edge has no weight, as shown in Fig. 5.

In Fig. 5, the topology network structure contains three elements: node, edge and weight. The node element refers to the reason for the generation and end of logistics. In the network structure, the attributes of the node include node location, node quantity, node storage capacity, cost, dealer demand, etc., and the node location includes the manufacturer location, distribution center location, transportation node location and dealer location. Edge refers to the basis of logistics realization between nodes. Two nodes are connected, that is, edge. If there are multiple transportation modes at nodes, multiple edges can be formed between nodes. Weight is an attribute on the edge, which indicates the strength difference of the item action of the edge of different nodes. The attributes of weight are transportation capacity, transportation time and transportation cost. In the vehicle logistics multimodal transport topology network, which nodes are selected and the connection mode between the edges need to be solved by using the model or algorithm, and the final solution result is the optimization result. Therefore, the research also needs to build a transportation scheme optimization model based on the optimal profit. The determination of the vehicle logistics transportation scheme is divided into two aspects: transportation route and transportation mode, mainly including transportation cost, time constraint and capacity constraint. The research assumes that the demand of a dealer is provided by only one manufacturer, all manufacturers, distribution nodes, dealers' locations, distances between nodes, node capacities, logistics costs between nodes, and dealer demand are known, and large vehicles are not considered for transport vehicles. Based on the above assumptions, the cost calculation formula for a transport route in the vehicle logistics multimodal transport network is shown in formula (11).

11 $$C={L}_{kj}{V}_{kjs}{U}_{s}+{L}_{Rkj}{V}_{kjs}{U}_{Rs}+{L}_{Wkj}{V}_{kjs}{U}_{Ws}+{L}_{Gkj}{V}_{kjs}{U}_{Gs}+{V}_{kjs}\left({U}_{k}+\sum_{r}^{R}{U}_{r}+\sum_{w}^{W}{U}_{w}+\sum_{d}^{D}{U}_{d}\right)$$

In formula (11), ${L}_{kj}$ means the highway transportation mileage in the transportation mileage from the manufacturer $k$ to the dealer $j$, ${V}_{kjs}$ is the small vehicle transportation volume from the manufacturer $k$ to the dealer $j$, and ${U}_{s}$ is the unit cost of small vehicle highway transportation. ${L}_{Rkj}$ is the transportation mileage from the manufacturer $k$ to the dealer $j$, ${U}_{Rs}$ means the unit cost of small vehicle railway transportation, ${U}_{Ws}$ is the unit cost of small vehicle waterway transportation, ${U}_{Gs}$ is the unit cost of small vehicle short-distance connection transportation, and ${U}_{k}$ refers to the unit cost of vehicle pickup and reshipment at the main engine plant. ${U}_{r}$ is the unit cost of vehicle pickup and reshipment at the railway station, ${U}_{w}$ is the unit cost of vehicle pickup and reshipment at the port, and ${U}_{d}$ is the unit cost of vehicle pickup and reshipment at the distribution center. In addition to the transportation cost calculation formula, the income calculation formula is also established, as shown in formula (12).

12 $$E={L}_{kj}{V}_{kjs}{U}_{s}+{L}_{Rkj}{V}_{kjs}{E}_{Rs}+{L}_{Wkj}{V}_{kjs}{E}_{Ws}+{L}_{Gkj}{V}_{kjs}{E}_{Gs}+{V}_{kj}\left({E}_{k}+\sum_{r}^{R}{E}_{r}+\sum_{w}^{W}{E}_{w}+\sum_{d}^{D}{E}_{d}\right)$$

In formula (12), ${E}_{Rs}$ refers to the income of small vehicle railway transportation unit, ${E}_{Ws}$ is the income of small vehicle waterway transportation unit, ${E}_{Gs}$ is the income of small vehicle short-distance connection transportation unit, ${E}_{k}$ is the income of main engine plant pick-up and reshipment unit, ${E}_{r}$ is the income of railway station pick-up and reshipment unit, ${E}_{w}$ is the income of port pick-up and reshipment unit, and ${E}_{d}$ is the income of distribution center pick-up and reshipment unit. The cost and income of a single line can be obtained through formula (11) and formula (12), and the maximum profit objective function of the whole network of vehicle logistics multimodal transport network can be obtained by upward derivation, as shown in formula (13).

13 $$Max{P}_{a}={E}_{a}-{C}_{a}$$

In formula (13), the whole network transportation cost calculation formula ${C}_{a}$ is shown in formula (14).

14 $${C}_{a}=\sum_{k}^{K}\sum_{j}^{J}\left[{L}_{kj}{V}_{kjs}{U}_{s}+{L}_{Rkj}{V}_{kjs}{U}_{Rs}+{L}_{Wkj}{V}_{kjs}{U}_{Ws}+{L}_{Gkj}{V}_{kjs}{U}_{Gs}+{V}_{kjs}\left({U}_{k}+\sum_{r}^{R}{U}_{r}+\sum_{w}^{W}{U}_{w}+\sum_{d}^{D}{U}_{d}\right)\right]$$

Accordingly, the total revenue ${E}_{a}$ of the transportation network can be expressed by formula (15).

15 $${E}_{a}=\sum_{k}^{K}\sum_{j}^{J}{L}_{kj}{V}_{kjs}{U}_{s}+{L}_{Rkj}{V}_{kjs}{E}_{Rs}+{L}_{Wkj}{V}_{kjs}{E}_{Ws}+{L}_{Gkj}{V}_{kjs}{E}_{Gs}+{V}_{kj}\left({E}_{k}+\sum_{r}^{R}{E}_{r}+\sum_{w}^{W}{E}_{w}+\sum_{d}^{D}{E}_{d}\right)$$

**Figure 6:**
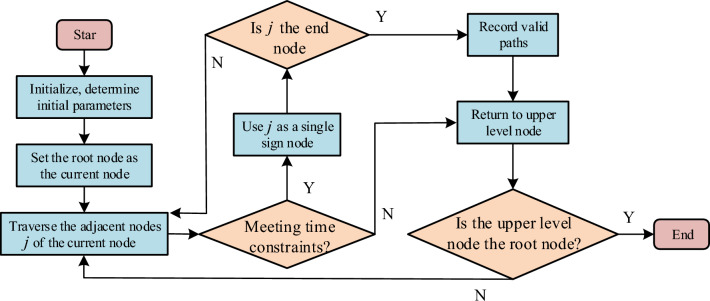
The graph traverses the algorithmic flow.

After the establishment of objective function, it is also necessary to supplement the corresponding constraints. In practice, vehicle logistics transportation needs to meet two constraints, namely, capacity constraints and time constraints. Capacity constraints include distribution center capacity constraints, railway station capacity constraints and water port capacity constraints. Time constraints refer to the longest transportation time acceptable to dealers. In the actual transportation network, there are many paths that meet the requirements of both manufacturers and dealers. At the same time, there must be paths that cannot meet the time constraints. If the path cannot meet the constraints, it will not be selected regardless of the profit space of the path. Therefore, when solving the path, it is necessary to eliminate these paths that cannot meet the constraints, and find the path with the largest profit space in the remaining feasible paths. Therefore, a restricted graph traversal algorithm is proposed to screen the feasible paths in all paths. First is to initialize the data, and input the basic data such as transportation effectiveness into the algorithm model. Second is to set the root node as the current node. Third is to start from the current node and traverse all nodes connected to the current node, And judge that the traversal time of the root node along the path is less than or equal to the time constraint condition, then update the traversal node to the current node until it is the end node. The fourth step is to record and retain all valid paths. The fifth step is to backtrack and return to the upper node. If it is the root node, the algorithm will be ended. If it is not the root node, the third step will be returned, as shown in Fig. 6.

**Figure 7:**
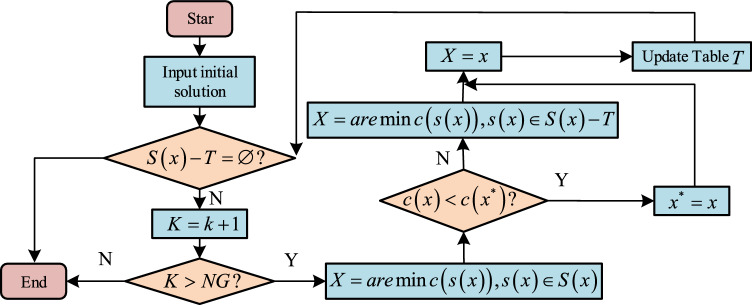
Tabu search flow.

Through the above algorithm, the paths that cannot meet the constraints can be excluded, and the path that meets the maximum profit space can be found in the remaining paths for optimization. In the vehicle logistics multimodal transport network, there are many nodes with high complexity and complex operation. In the face of such optimization problems with high complexity and difficult calculation, heuristic algorithms are usually used to solve them. However, the general heuristic algorithms have their own defects. For example, genetic algorithms are applicable to local optimization, while global optimization is poor. The parameter selection of the simulated annealing algorithm is difficult and the calculation is complicated, while Tabu search is applied to path optimization, which can not only avoid the algorithm from falling into local optimization, but also simplify the calculation process and reduce the number of iterations of the algorithm. Therefore, the objective function is proposed to solve with taboo rule. The Tabu rule means that the algorithm is prohibited from performing repeated work in the iterative process. The existence of Tabu rules can effectively avoid the algorithm falling into local optimization in the iterative optimization process. Tabu search algorithm can improve the operation efficiency and avoid repeated search by setting a Tabu table for the tracking object. However, in practical application, Tabu search needs to input an initial solution to improve the calculation speed of the algorithm. The higher the initial solution quality, the better the algorithm calculation effect. Therefore, the original transportation scheme of a logistics enterprise is studied as the initial solution to enhance the algorithm operation effect. The steps of Tabu search algorithm are as follows: the first step is to input the initial solution of logistics transportation route; the second step is to take all feasible routes as the search field for centralized search; the third step is to make the initial Tabu list empty; the fourth step is to replace the distribution of dealers; the graph traversal algorithm eliminates the paths that do not meet the time constraints. In Tabu search algorithm, only the capacity of nodes is needed, As a constraint, calculate the profit space of each feasible path. The fifth step is to store the obtained feasible solution in the Tabu table and update it. The last step is to determine whether the termination rule is met. If it is met, the algorithm will end. If not, return to the fourth step to continue searching, as shown in Fig. 7.

Tabu search needs to design the key elements of the algorithm in solving. The key elements of the algorithm include the initial solution, step size, evaluation function, etc. Some key factors have been designed. The Tabu step size and evaluation function are designed as follows. The Tabu step size is set to *n*, an integer no more than 10. The purpose of the research is to improve the profit space of vehicle logistics enterprises, and the whole network profit target value in the vehicle logistics multimodal transport network is taken as the evaluation function.

## Simulation experiment results analysis

The main content of this chapter is the research and analysis of the experimental results. The first section is the performance comparison of Tabu search and other methods, and the second section is the analysis of the optimization results of the model.

### Algorithm performance results comparative analysis

In the taboo search algorithm, parameters such as initial feasible solution, neighborhood movement, taboo table length, and number of neighborhoods are important parameters. The study presents the structure of the taboo search algorithm solution in the form of a set, with the corresponding path of the dealer as the element of the solution set. The initial feasible solution is the initial transportation plan of the logistics enterprise. The rules for formulating the neighborhood structure of the solution are as follows: based on the initial solution, represent the path that meets the distributor's time requirements, replace the corresponding distributor's delivery path in the solution set, form a neighborhood solution equal to the number of distributors, and then constrain the neighborhood solution to remove some solutions to select a candidate solution set. The shorter the taboo step size, the less memory is occupied by the device, and the search range is larger. However, it is prone to getting stuck in local optima. If the taboo step size is too long, it can lead to a decrease in the efficiency of the algorithm. The evaluation function of taboo search algorithm is enterprise profit, and the amnesty criterion needs to be judged based on the objective function and the cycle of the solution. According to the parameter setting rules of the taboo search algorithm mentioned above, the parameters of the taboo search algorithm are shown in Table 1.

**Table 1:** Taboo search algorithm parameters.

Name	Value	Name	Value
Maximum iteration times	190	Neighbor structure	57
Taboo step length	6	Initial solution	Original transportation route
Number of distributors	57	–	–

"–" means that the form has no fill.

**Figure 8:**
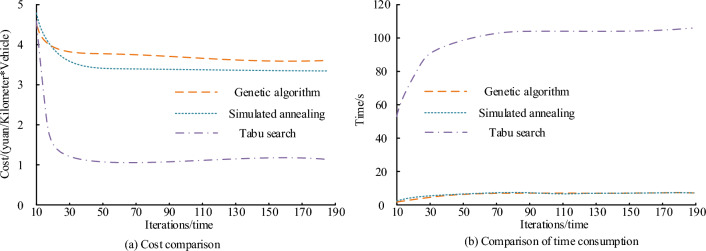
Comparison of algorithm time and cost results.

The taboo search algorithm, simulated annealing method, and genetic algorithm are commonly used heuristic algorithms for solving optimization problems. In order to verify the effectiveness of the taboo search algorithm in logistics transportation route optimization, the performance of the three algorithms was compared using Matlab 2016b. Govindan et al. designed a simulated annealing algorithm for logistics path optimization, Wang et al. designed a genetic algorithm for optimizing network node paths^19,20^. When comparing the above algorithms, the study sets the parameters of simulated annealing method and genetic algorithm based on the parameters in references. The convergence results of the algorithm's time consumption and cost are shown in Fig. 8.

**Figure 9:**
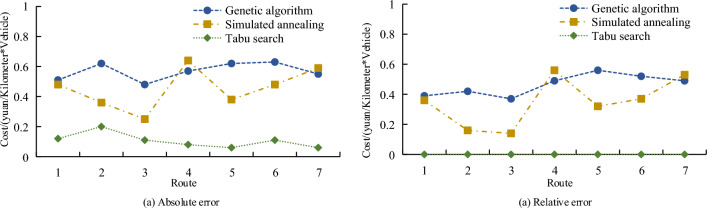
Algorithm error comparison.

Figure 8a shows the cost calculation convergence results. Tabu search algorithm completes the convergence first when calculating the transportation cost. When iteration is 18, Tabu search completes the convergence. After the convergence, the cost of Tabu search is 1.2 yuan/km × per set, and the genetic algorithm completes the convergence after 12 iterations. After the convergence, the cost of the genetic algorithm is 3.8 yuan/km × per set, the simulated annealing method converges after 35 iterations. After convergence, the cost of the simulated annealing method is 3.2 yuan/km × per set. Figure 8b shows the comparison of the calculation time. Tabu search algorithm calculation time is much higher than that of other methods. The operation time of Tabu search algorithm is more than 100 s, and the calculation time of genetic algorithm and simulated annealing method is less than 10 s. The calculation error results of the three algorithms in different routes are shown in Fig. 9.

**Figure 10:**
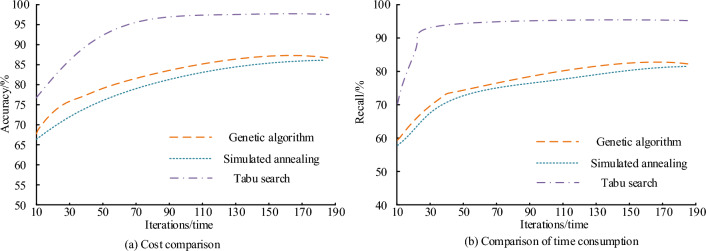
Comparison of accuracy and recall rates.

Figure 9a absolute error comparison of the three algorithms shows that the absolute error of Tabu search algorithm is much lower than that of other methods. The maximum error of genetic algorithm is 0.63 yuan/km × per set, that of simulated annealing method is 0.64 yuan/km × per set, and that of Tabu search algorithm is 0.20 yuan/km × per set. Figure 9b shows the relative errors of other methods relative to Tabu search algorithm. Average relative error of genetic algorithm is higher. The comparison results of the accuracy and recall rates of the three algorithms are shown in Fig. 10.

Figure 10a shows the accuracy comparison. The accuracy of Tabu search algorithm is much higher than that of other methods. At the beginning, the accuracy rate of Tabu search is 77%, after convergence, the accuracy rate of Tabu search is about 95%, while that of genetic algorithm is 68% at the beginning, and after convergence, the accuracy rate of genetic algorithm is about 81%. Figure 10b shows the comparison of the recall rates. Comparison results are similar to the accuracy rates. The recall rate of Tabu search is much higher than that of genetic algorithm and simulated annealing method. After convergence, the recall rate of Tabu search algorithm is about 92%, that of genetic algorithm is about 78%, and that of simulated annealing method is about 77%. In NGSIM dataset, receiver operating characteristic curve (ROC) curves of three algorithms are analyzed below.

**Figure 11:**
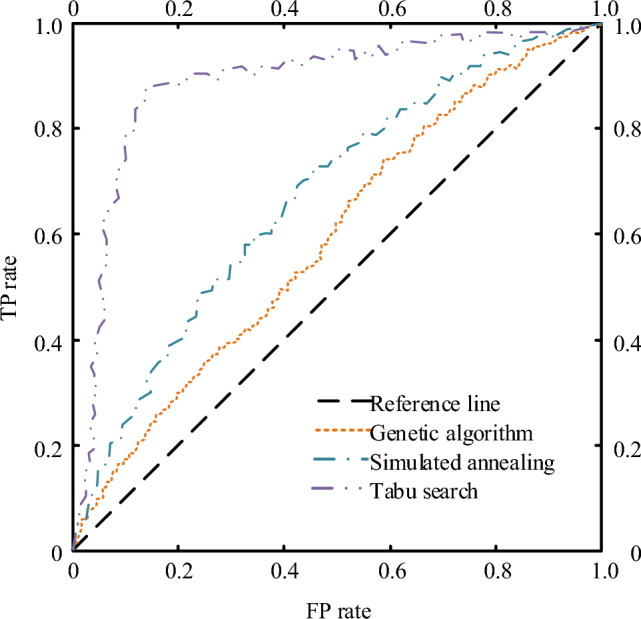
Comparison results of receiver operating characteristics of three algorithms.

**Figure 12:**
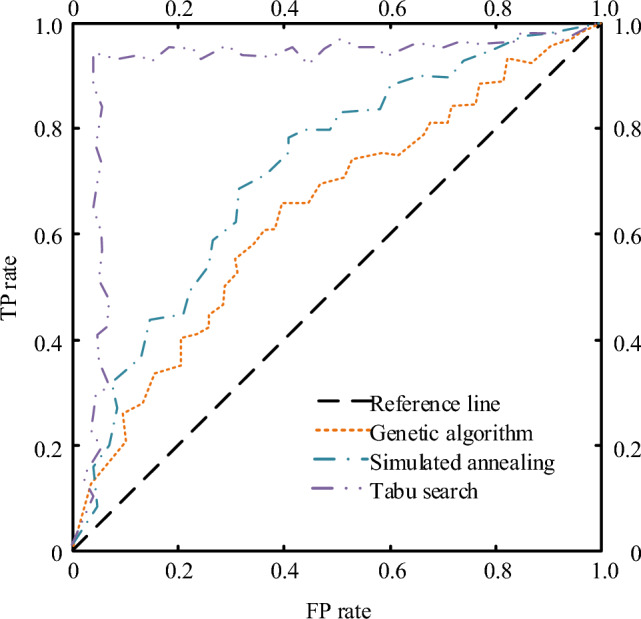
ROC comparison of three algorithms in NGSIM dataset.

Figure 11 shows the ROC curve comparison results of the three algorithms on the NGSIM dataset. The TP rate and FP rate of Tabu algorithm can reach more than 90%, it can be seen that the product under the curve of Tabu search is much larger than the area under the curve of other methods, indicating that the optimization performance of Tabu search on the transportation path in the NGSIM dataset is significantly stronger than that of other methods. To increase the credibility of the experimental data, the study also compared and analyzed the ROC curves of the three algorithms in the high D data set, and the results are shown in Fig. 12.

Figure 12 shows the ROC curve comparison results of the three algorithms on the high D dataset. It can be seen that the area under the curve of Tabu search is still much higher than that of other methods, indicating that in the transportation path optimization, the performance of Tabu search is better than that of the genetic algorithm and the simulated annealing method, and the result is not accidental.

### Logistics intermodal route optimization results analysis

After studying and determining the optimization effect of Tabu search algorithm in the logistics transportation route, taking the logistics transportation route of logistics company X as an example, the vehicle logistics multimodal transportation route was optimized. The company's initial transport route is mainly by road transport, a small number of sections used by railway transport, without water transport, road transport accounts for 87% of the total route. The company has two manufacturers, which are located in region a and region B respectively. At the same time, there are 57 dealers in all regions. The company has five distribution centers and is expected to add four new distribution centers. The company's transportation costs and revenues are shown below (see Table 2).

**Table 2:** X company transportation information.

Mode of transport	Transportation profile (yuan/km × vehicle)		Profit from recovery (yuan/vehicle)		
	Large vehicles	Light duty vehicle	Road	Railway	Water
Railway transportation	1.80	1.81	10	0	10
Road transport	1.03	0.81	0	10	10
Water transportation	1.87	2.14	10	10	0
Short distance connection	0.57	0.29	/	/	/

**Figure 13:**
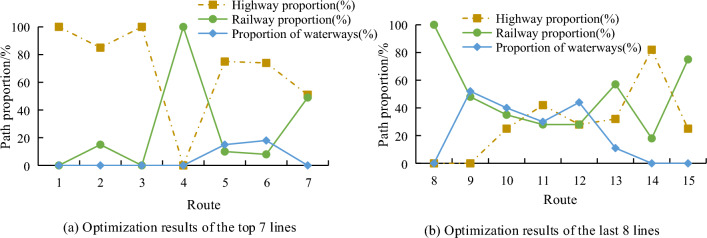
Optimization results of transportation plan.

Since company X plans to build four new distribution centers, the improved *P *median model is used to determine the location of distribution centers. Lingo software is used to complete the programming calculation of the location model of distribution centers, which will not be detailed. After determining the location of the new distribution center, the Tabu search algorithm was used to optimize the transportation scheme of the whole vehicle logistics part of the intermodal route of X company, and 15 routes of all routes were selected and drawn as Fig. 13.

**Figure 14:**
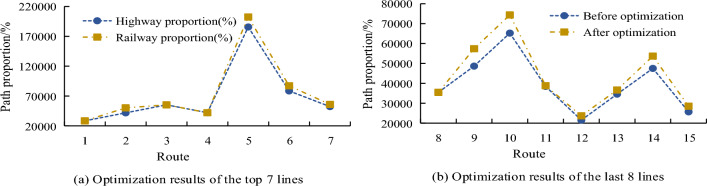
Profit changes after optimization.

Figure 13a shows the first 7 of the 15 transportation routes, and Fig. 13b shows the last 8 of the 15 transportation routes. It can be seen that after optimization, routes 1, 3, 4 and 8 still use only one mode of transportation, while routes 2, 7, 9, 14 and 15 are no longer a single mode of transportation after optimization, and the rest of the routes use three modes of transportation after optimization. The study also compared the profit changes of the route before and after optimization, and the results are shown in Fig. 14.

Figure 14a shows the first seven of the 15 transportation routes, and Fig. 14b shows the last eight of the 15 transportation routes. It can be seen that only the profits of Route 1, route 3, Route 4 and route 8 remain unchanged before and after optimization, consistent with the changes in transportation mode before and after optimization. The profits of other routes have increased, of which route 9 has the largest increase in profits. After optimization, the profits of this route have increased by 18%. In order to determine the planning route effect of the taboo search algorithm, the profit changes of different routes before and after planning were compared, and the results are shown in Table 3.

**Table 3:** The optimization effect comparison of different routes.

Algorithm	Time (s)	Maximum deviation (%)	Average deviation (%)
SSA	5.64–7.24	94.5	26.4
GA	4.38–8.54	89.6	25.1
SSA-GA	2.25–6.38	83.7	22.3
Local search	4.97–9.84	92.4	32.8
Tabu	1.26–3.56	0	0

In Table 3, you can see that the simulated annealing operation time up to 7.24 s, genetic algorithm operation time to 8.54 s, based on genetic algorithm to improve simulated annealing method time is 6.38 s, local search algorithm time to 9.84 s, and the Tabu algorithm time only 3.56 s, with Tabu algorithm as the optimal solution, the maximum deviation of simulated annealing method is 94.5%, the average deviation of the local search algorithm, 32.8%.

## Discussion

A study has proposed an optimization method for vehicle logistics intermodal transportation routes based on taboo search algorithm. By optimizing the transportation mode and route of vehicle logistics, the aim is to reduce logistics transportation costs and increase the profit space of logistics enterprises. The optimization of vehicle logistics intermodal transportation routes is an important issue facing the current logistics industry. With the development of logistics enterprises and the adjustment of relevant regulations, the profit space of vehicle logistics enterprises has been further compressed. By building a multimodal transportation network for vehicle logistics, not only can the advantages of railway and waterway transportation be fully utilized, logistics costs can be reduced, but also the profit space of logistics enterprises can be increased. The study uses graph traversal algorithm to screen feasible paths in the multimodal transportation network of vehicle logistics. The purpose of this step is to find a path that has good benefits and meets the needs for further optimization. Then, the tabu search algorithm is used to solve the optimization problem of the vehicle logistics multimodal transportation route model. Tabu search algorithms can effectively explore the search space and avoid falling into local optima. The research results indicate that the performance of taboo search algorithm in solving route optimization problems is superior to genetic algorithm and simulated annealing method. The optimal route cost obtained through taboo search algorithm is 1.2 yuan/kilometer * per unit, and it performs better than other algorithms on the NGSIM dataset.

## Conclusion

In order to increase the transportation cost and revenue of logistics in vehicle logistics, and increase the profit space of logistics enterprises, a vehicle logistics multimodal transport route optimization scheme using Tabu search is proposed. The scheme establishes the vehicle logistics multimodal transport network structure, and uses graph traversal algorithm to screen the effective path in the intermodal transport network, and then uses Tabu search to optimize the effective path in the vehicle logistics multimodal transport network. The results showed that the performance of Tabu search algorithm in logistics transportation route optimization was better than other methds. The convergence cost of Tabu search algorithm was 1.2 yuan/km × per set, and the cost of the other two algorithms was more than 2.0 yuan/km × per set. The running time of Tabu search was higher than that of genetic algorithm and simulated annealing algorithm. The running time of Tabu search was about 100 s, and the running time of the other two algorithms was less than 10 s. The calculation error of Tabu search algorithm was lower. The maximum absolute error of Tabu search algorithm was 0.2 yuan/km × per set, the minimum absolute error of genetic algorithm was 0.42 yuan/km × per set, and the minimum absolute error of simulated annealing method was 0.23 yuan/km × per set. Tabu algorithm was effective for the optimization of vehicle logistics intermodal routes. Of the 15 routes, only 4 routes were not optimized, and the remaining routes were optimized. The profits of all the optimized intermodal routes increased, and the profits of Route 9 increased by 18%. This paper studies and constructs the vehicle logistics multimodal transport route optimization model, and uses Tabu algorithm to solve the model, which effectively improves the profit space of vehicle logistics enterprises. However, in the process of establishing the model, the loading capacity of transportation vehicles is not comprehensively considered, so the problem can be further refined.

## Data Availability

All data generated or analysed during this study are included in this published article.
